# Multiple *Scedosporium apiospermum* abscesses in a woman survivor of a tsunami in northeastern Japan: a case report

**DOI:** 10.1186/1752-1947-5-526

**Published:** 2011-10-25

**Authors:** Yutaka Nakamura, Yu Utsumi, Naomi Suzuki, Yoshio Nakajima, Okinori Murata, Nobuhito Sasaki, Hiroo Nitanai, Hiromi Nagashima, Shinya Miyamoto, Jun Yaegashi, Tomoki Hatakeyama, Yoshihiro Shibano, Kyoko Yarita, Katsuhiko Kamei, Toshihide Nakadate, Shigeatsu Endo, Yasuo Terayama, Kohei Yamauchi

**Affiliations:** 1 Division of Pulmonary Medicine, Allergy, and Rheumatology, Department of Internal Medicine, Iwate Medical University School of Medicine, 19-1 Uchimaru, Morioka, 0208505, Japan; 2 Department of Pulmonary Medicine, Iwate Prefectural Miyako Hospital, 1-11-26 Sakikuwagasaki, Miyako, 0270096, Japan; 3 Department of Internal Medicine, Saiseikai Iwaizumi Hospital, 19-1 Nakaya, Iwaizumi aza, Iwaizumi town, Shimoheigun, 0270501, Japan; 4 Medical Mycology Research Center, Chiba University, 1-8-1 Inohana, Chiba, 2608673, Japan; 5 Department of Critical Care Medicine, Critical Care and Emergency Center, Iwate Medical University School of Medicine, 19-1 Uchimaru, Morioka, 0208505, Japan; 6 Division of Neurology and Gerontology, Department of Internal Medicine, Iwate Medical University School of Medicine, 19-1 Uchimaru, Morioka, 0208505, Japan

## Abstract

**Introduction:**

*Scedosporium apiospermum *is increasingly recognized as a cause of localized and disseminated mycotic infections in near-drowning victims.

**Case presentation:**

We report the case of a 59-year-old Japanese woman who was a survivor of a tsunami in northeastern Japan and who had lung and brain abscesses caused by *S. apiospermum*. Initially, an aspergillus infection was suspected, so she was treated with micafungin. However, computed tomography scans of her chest revealed lung abscesses, and magnetic resonance images demonstrated multiple abscesses in her brain. *S. apiospermum *was cultured from her bronchoalveolar lavage fluid, and antimycotic therapy with voriconazole was initiated. Since she developed an increase in the frequency of premature ventricular contractions, an adverse drug reaction to the voriconazole was suspected. She was started on a treatment of a combination of low-dose voriconazole and liposomal amphotericin B. After combination therapy, further computed tomography scans of the chest and magnetic resonance images of her brain showed a demarcation of abscesses.

**Conclusions:**

Voriconazole appeared to have a successful record in treating scedosporiosis after a near drowning but, owing to several adverse effects, may possibly not be recommended. Thus, a combination treatment of low-dose voriconazole and liposomal amphotericin B may be a safe and effective treatment for an *S. apiospermum *infection. Even though a diagnosis of scedosporiosis may be difficult, a fast and correct etiological diagnosis could improve the patient's chance of recovery in any case.

## Introduction

Tsunami lung occurs when a person who is overwhelmed by tsunami waves inhales saltwater contaminated by mud and microorganisms. Some microorganisms regarded as harmless saprophytes are, with increasing frequency, being reported to cause serious or lethal infections, even in immunocompetent individuals. *Scedosporium apiospermum *is increasingly recognized as a cause of localized and disseminated mycotic infections in near-drowning victims. This ubiquitous fungus is present in soil, manure, sewage, and polluted waters. Here, we describe the case of a patient with both lung and brain abscesses caused by *S. apiospermum *and the therapeutic approach used for patients with tsunami lung associated with near drowning.

## Case presentation

A previously healthy 59-year-old Japanese woman had been swept away by the tsunami that struck the Sanriku coast in northeastern Japan. She aspirated saltwater contaminated with soil and heavy oil, which had run out from the capsized ships. She swam back ashore and reached a shelter at a gymnasium by herself. She was transferred to a neighboring hospital, where she developed respiratory failure within a few hours of admission, was intubated, and was placed on a respirator. Management with positive end-expiratory pressure, fluid restriction, and broad-spectrum antibiotics did not lead to any improvement in her pneumonia. She was referred to our hospital five days after she had almost drowned. Upon admission (on day six), she had a fever of 39°C, a blood pressure of 142/92 mm Hg, and a tachycardia of 112 beats per minute. There was no evidence of trauma or fractures anywhere on her body. Auscultation of the chest revealed some crackles in both lungs. Her heart sounds were normal, and the results of an abdominal examination were normal. Her hemoglobin level was 10.0 g/dL, her platelet count was 27.4 × 10^10^/L, and her white cell count 12.9 × 10^9^/L, and she had a neutrophilia of 11.7 × 10^9^/L.

**Figure 1:**
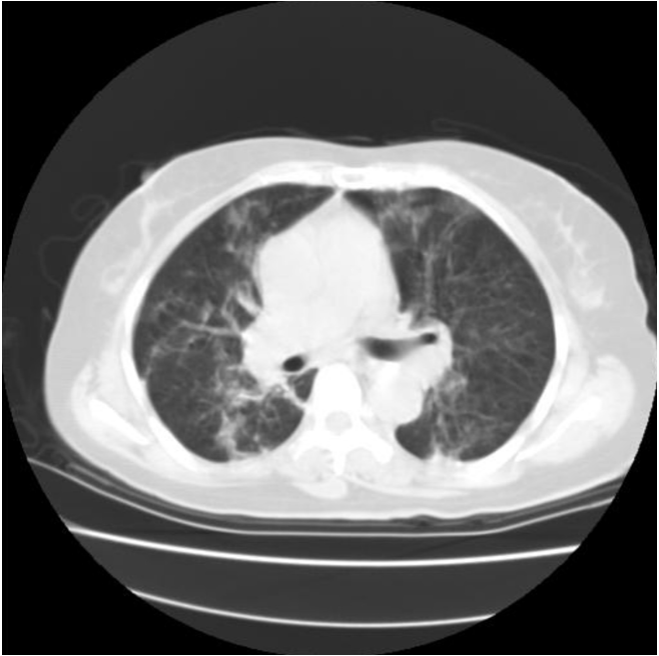
**The chest computed tomography scan from admission shows that nodular shadows and filtrates are scattered in both lungs**.

**Figure 2:**
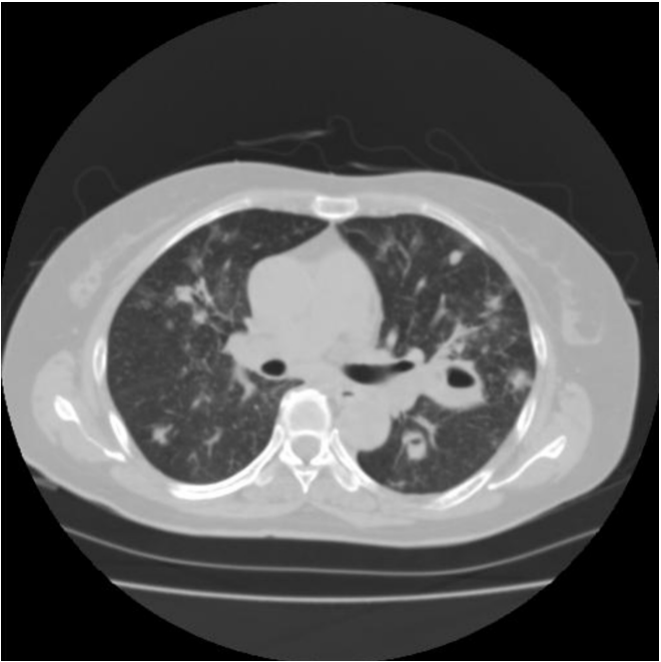
**The chest computed tomography scan from day 28 shows a cavity in the left upper lobe, which has a thick wall and is surrounded by consolidation**.

**Figure 3:**
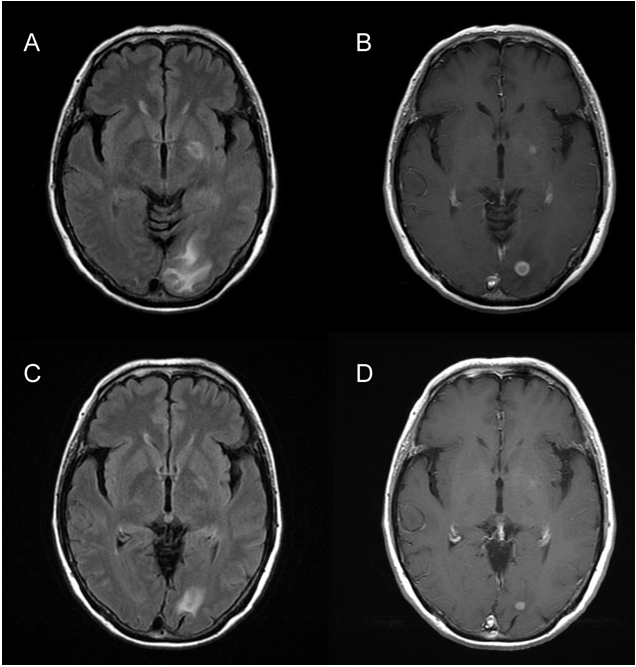
**Magnetic resonance imaging of the brain. **The axial fluid-attenuated inversion-recovery (FLAIR) image shows multiple hyperintensity lesions in the left occipital white matter and in the left internal capsule (a). The post-contrast T1-weighted image shows multiple rounded lesions with peripheral capsular enhancement in the corresponding area (b). Almost four weeks later, multiple hyperintensity lesions in the left occipital white matter and the left internal capsule (c) as well as multiple enhanced lesions (d) are diminished in size and intensity.

**Figure 4:**
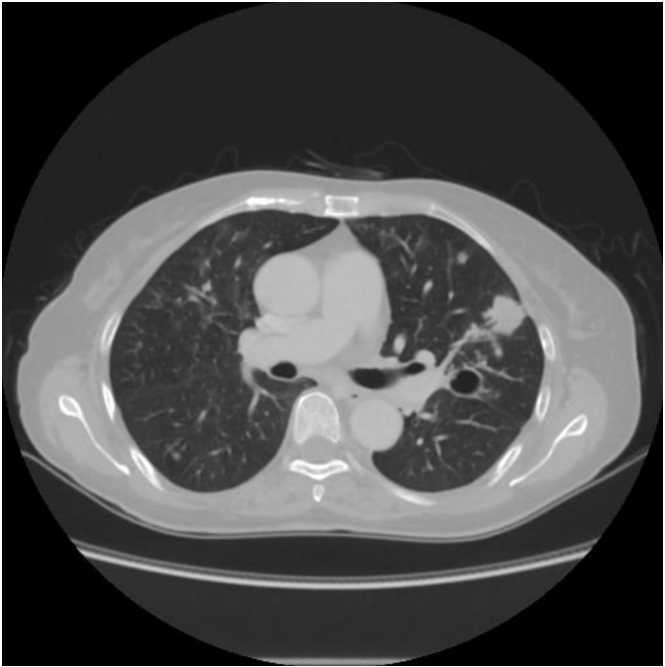
**The chest computed tomography scan from day 93 shows the cavity at the left upper lobe, which has become reduced in size**.

Her serum albumin was reduced to 23 g/L, and her alkaline phosphatase was increased to 357U/L (normal range of 99 to 340U/L). Her C-reactive protein (CRP) was raised to 185 mg/L. Serum urea, creatinine, electrolytes, glucose, and coagulation were within normal reference ranges. A urine analysis showed normal findings. A 12-lead electrocardiogram showed sinus tachycardia. Computed tomography (CT) scanning of her head was normal and that of her chest revealed infiltration and nodular lesions in both sides of her lungs (Figure 1). Blood cultures were taken from our patient after admission and showed no growth. A bronchoalveolar lavage (BAL) was performed by inserting a flexible fiberoptic bronchoscope. The BAL fluid contained oil, sand, and small pieces of wood. Tsunami lung was suspected, and, in addition to an antifungal treatment, broad-spectrum antibiotics were used, starting with 500 mg of carbapenem intravenously three times a day (t.i.d.), 500 mg of pazufloxacin intravenously twice a day (b.i.d.), 150 mg of micafungin intravenously once a day (s.i.d.), and steroid pulse therapy. Nevertheless, a follow-up CT scan of the lung on day 28 showed multiple nodular lesions of the bilateral lung and a cavity in the upper left lobe (Figure 2), and the level of (1→3)-β-D-glucan (from 11.7 to 110 pg/mL) had increased. Therefore, trimethoprim-sulfamethoxazole (trimethoprim 4.8 mg/kg t.i.d.) and 500 mg of vancomycin intravenously four times a day were added to the treatment. At this time, a new BAL was examined, and although BAL fluid cultures for common and anaerobic bacteria were also negative, filamentous fungi, which seemed to be the cause in this case, were seen. On the basis of these results, the antimycotic therapy was switched to voriconazole (initially 6 mg/kg b.i.d. followed by 4 mg/kg b.i.d.). On day 36, our patient developed a palpitation, and the frequency of premature ventricular contractions (PVCs) increased, although electrolyte imbalance and high concentrations of voriconazole in the plasma were not examined. An adverse drug reaction of voriconazole was suspected, and the drug was withdrawn. Our patient was started on liposomal amphotericin B (2.5 mg/kg s.i.d.) along with trimethoprim-sulfamethoxazole, vancomycin, and 500 mg of levofloxacin intravenously s.i.d. After the start of liposomal amphotericin B therapy, our patient's condition ameliorated and she was weaned successfully from mechanical ventilation. Magnetic resonance imaging of her head on day 63 revealed multiple brain abscesses (Figure 3a,b), but the results of her physical and neurological examinations were unremarkable, except for the presence of a mild fever. A lumbar puncture was performed but showed no white cells and no identifiable organisms on a Gram stain or upon a culture of the cerebrospinal fluid. On day 69, the diagnosis of *S. apiospermum *infection was confirmed by polymerase chain reaction and DNA sequencing of cultured filamentous fungi obtained from the previous BAL specimen. Since the isolate was considered susceptible to voriconazole (minimum inhibitory concentration [MIC] of 0.09 μg/mL), our patient was started on low-dose intravenous voriconazole (initially 6 mg/kg b.i.d. followed by 2 mg/kg b.i.d.) with liposomal amphotericin B (MIC of 1 μg/mL), which showed a relatively low MIC against *S. apiospermum*. After 15 days of treatment with a combination of voriconazole and liposomal amphotericin B, further magnetic resonance imaging of the head showed a demarcation of abscesses in the brain on day 88 (Figure 3c,d). After the start of a combination therapy, the low-grade fever recurred, and the levels of both CRP and (1→3)-β-D-glucan declined. On day 93, a chest CT showed the size of the cavity, the consolidation at the left upper lobe had reduced in size, and many of the nodular shadows in the lung fields had disappeared (Figure 4). Oral administration of voriconazole was substituted with intravenous voriconazole and included the addition of oral levofloxacin 500 mg and clarithromycin 400 mg. The voriconazole serum levels, checked regularly, were found to be within therapeutic limits. No recurrence was noted, and our patient was transferred to a hospital in her hometown 96 days after treatment had been initiated.

## Discussion

*S. apiospermum *generally shows a high propensity to cause central nervous system (CNS) infections. The mode of *S. apiospermum *invasion and its subsequent expansion to the CNS remains ambiguous. Probable pathophysiological mechanisms of *S. apiospermum *infection after a near drowning include a local spread from sites near the brain, such as the paranasal sinuses [1] or cribiform plate [2], and a hematogenous spread from the lungs.

Voriconazole has shown efficacy in CNS-related scedosporiosis refractory to treatment with other antifungal agents, and several case reports and open-label clinical studies have provided *in vivo *evidence of the efficacy and tolerability of voriconazole as a method of therapy in patients with *Scedosporium *infections [3-5]. Another study describes voriconazole concentrations that are higher in cerebrospinal fluid than in plasma in a female patient [6]. Though generally well tolerated, voriconazole therapy may be associated with adverse events like transient visual disturbances, hepatotoxicity, skin rashes [7,8], and life-threatening ventricular arrhythmia [9]. In our patient, voriconazole appeared to be effective but was accompanied by PVCs and so was discontinued. However, *in vitro *susceptibility studies have shown that *S. apiospermum *isolates are susceptible to voriconazole and appear to have variable susceptibility to amphotericin B [5,10-13]. In our patient, susceptibility testing according to the methods of the Clinical and Laboratory Standards Institute was performed; the testing showed that the MIC for amphotericin B was relatively low and in agreement with the previous results that had higher MICs for flucytosine, fluconazole, itraconazole, miconazole, and micafungin. Because enhanced antifungal activity has been demonstrated *in vitro *for combinations of amphotericin B plus azoles [14,15], we had decided to add low-dose voriconazole to amphotericin B while using electrocardiographic monitoring. After voriconazole therapy was reinstated in a low dose, adverse events, such as PVCs, were not seen. The synergistic interaction on our patient can be evaluated even though low-dose voriconazole was administered.

## Conclusions

In our patient, there was no evidence of fungal infections during the first month after her near-drowning experience. In addition, repeated blood cultures remained negative for fungi. Initially, aspergillosis was suspected, so our patient was treated with micafungin, although aspergillosis after near drowning seems to be rarer than scedosporiosis [12]. Another victim of the tsunami, who was also referred to our hospital, was a Japanese woman with *Scedosporium prolificans*. Before the fungus was diagnosed, she received treatments of antibiotics, steroids, and extracorporeal membrane oxygenation. She continued to deteriorate because of infection and succumbed to septic shock. In this fatal case, the lack of defined abscesses and specific symptoms of fungi infection prevented antifungal treatment from being administered. Because the detection of *Scedosporium *was confined to the BAL from the lung abscess, it was obviously too late for a successful antifungal therapy. Katragkou and colleagues [12] reported that the median 'time to diagnosis of *Scedosporium *infection' was 28 days. This could be attributed to the low sensitivity of routine culture methods. In our patient, since conidia had not formed, filamentous fungi were cultured repeatedly and, on day 64, were submitted to polymerase chain reaction and DNA sequencing.

The tsunami wiped out much of the medical infrastructure, and many medications were not available. Therefore, we propose that voriconazole be used empirically and very early in patients who have nearly drowned. When voriconazole should not be used because of adverse effects, the use of combined therapies can be a promising clinical approach for combating infections caused by multidrug-resistant fungi. A fast and correct etiological diagnosis could improve the patient's outcome in any case.

## Abbreviations

BAL: bronchoalveolar lavage; b.i.d.: twice a day; CNS: central nervous system; CRP: C-reactive protein; CT: computed tomography; MIC: minimum inhibitory concentration; PVC: premature ventricular contraction; s.i.d.: once a day; t.i.d.: three times a day.

## Consent

Written informed consent was obtained from the patient for publication of this case report and any accompanying images. A copy of the written consent is available for review by the Editor-in-Chief of this journal.

## Competing interests

The authors declare that they have no competing interests.

## Authors' contributions

All of the authors were involved in the conception of the case report, the data collection, and literature review as well as in writing the manuscript. YN was a major contributor in writing the manuscript. YU and NS performed the bronchoscopy. KY and KK identified the filamentous fungi as *S. apiospermum *and *S. prolificans*. All authors read and approved the final manuscript.
